# Sonobiopsy for minimally invasive, spatiotemporally-controlled, and sensitive detection of glioblastoma-derived circulating tumor DNA

**DOI:** 10.7150/thno.65597

**Published:** 2022-01-01

**Authors:** Christopher P. Pacia, Jinyun Yuan, Yimei Yue, Lu Xu, Arash Nazeri, Rupen Desai, H. Michael Gach, Xiaowei Wang, Michael R. Talcott, Aadel A. Chaudhuri, Gavin P. Dunn, Eric C. Leuthardt, Hong Chen

**Affiliations:** 1 Department of Biomedical Engineering, Washington University in St. Louis, Saint Louis, MO 63130, USA.; 2 Mallinckrodt Institute of Radiology, Washington University School of Medicine, Saint Louis, MO, 63110, USA.; 3 Department of Neurosurgery, Washington University School of Medicine, St. Louis, MO, 63110, USA.; 4 Andrew M. and Jane M. Bursky Center for Human Immunology and Immunotherapy Programs, Washington University School of Medicine, St. Louis, MO, 63110, USA.; 5 Department of Radiation Oncology, Washington University School of Medicine, Saint Louis, MO 63108, USA.; 6 Department of Pharmacology and Regenerative Medicine, University of Illinois at Chicago, Chicago, IL, 60612, USA.; 7 University of Illinois Cancer Center, Chicago, IL, 60612, USA.; 8 Division of Comparative Medicine, Washington University School of Medicine, Saint Louis, MO, 63110, USA.; 9 Department of Genetics, Washington University School of Medicine, St. Louis, MO, 63110, USA.; 10 Department of Computer Science and Engineering, Washington University in St. Louis, Saint Louis, MO 63130, USA.; 11 Siteman Cancer Center, Washington University School of Medicine, St. Louis, MO, 63110, USA.; 12 Department of Neuroscience, Washington University School of Medicine, Saint Louis, MO, 63110, USA.; 13 Center for Innovation in Neuroscience and Technology, Washington University School of Medicine, Saint Louis, MO, 63110, USA.

**Keywords:** Image-guided focused ultrasound, blood-brain barrier, blood-based liquid biopsy, glioblastoma mutation, droplet digital PCR

## Abstract

Though surgical biopsies provide direct access to tissue for genomic characterization of brain cancer, they are invasive and pose significant clinical risks. Brain cancer management via blood-based liquid biopsies is a minimally invasive alternative; however, the blood-brain barrier (BBB) restricts the release of brain tumor-derived molecular biomarkers necessary for sensitive diagnosis.

**Methods:** A mouse glioblastoma multiforme (GBM) model was used to demonstrate the capability of focused ultrasound (FUS)-enabled liquid biopsy (sonobiopsy) to improve the diagnostic sensitivity of brain tumor-specific genetic mutations compared with conventional blood-based liquid biopsy. Furthermore, a pig GBM model was developed to characterize the translational implications of sonobiopsy in humans. Magnetic resonance imaging (MRI)-guided FUS sonication was performed in mice and pigs to locally enhance the BBB permeability of the GBM tumor. Contrast-enhanced T_1_-weighted MR images were acquired to evaluate the BBB permeability change. Blood was collected immediately after FUS sonication. Droplet digital PCR was used to quantify the levels of brain tumor-specific genetic mutations in the circulating tumor DNA (ctDNA). Histological staining was performed to evaluate the potential for off-target tissue damage by sonobiopsy.

**Results:** Sonobiopsy improved the detection sensitivity of EGFRvIII from 7.14% to 64.71% and TERT C228T from 14.29% to 45.83% in the mouse GBM model. It also improved the diagnostic sensitivity of EGFRvIII from 28.57% to 100% and TERT C228T from 42.86% to 71.43% in the porcine GBM model.

**Conclusion:** Sonobiopsy disrupts the BBB at the spatially-targeted brain location, releases tumor-derived DNA into the blood circulation, and enables timely collection of ctDNA. Converging evidence from both mouse and pig GBM models strongly supports the clinical translation of sonobiopsy for the minimally invasive, spatiotemporally-controlled, and sensitive molecular characterization of brain cancer.

## Introduction

Brain cancer severely threatens human health due to their disruption of neurological function, poor prognosis, and substantial reduction in quality of life 1,2. At present, patients with glioblastoma (GBM), the most common primary brain tumor in adults, have a median survival time of 14 months from the time of diagnosis 3,4. Genomic characterizations of cancer are transforming clinical medicine, moving from the current model of population risk assessment and empirical treatment to individualized care based on molecular classification and targeted therapy 5-8. However, the lack of minimally invasive access to brain tumor specimens for genomic analysis precludes the molecular characterization of brain cancer over time and hinders the development of effective therapeutic approaches.

The two pillars of diagnostic management of malignant brain tumors are neuroimaging and surgically acquired tissue for pathology and genetic profiling. Current diagnostic evaluation typically relies on magnetic resonance imaging (MRI) and computed tomography to identify suspicious tumor lesions, followed by surgical resection or stereotactic biopsy for histological confirmation and genetic characterization. Because these procedures carry surgical risk 9, tissue biopsies cannot be performed for tumors at inoperable locations, or patients who are too ill to tolerate invasive procedures 10. Given the dynamic nature of these aggressive tumors, a routine interrogation to assess treatment response and cancer recurrence is critically needed. Yet, repeated tissue biopsies are often not feasible given the increased risk for complications and morbidity. In addition, tissue biopsies cannot capture the spatial heterogeneity because the genetic analysis is typically performed for a single tumor region 11.

Blood-based liquid biopsy (LBx) is a rapid and inexpensive way of obtaining clinically relevant information about the tumor without surgery 12. It is a promising approach for the diagnosis, molecular characterization, and monitoring of brain cancer by detecting circulating tumor-derived biomarkers, e.g., DNA, RNA, extracellular vesicles, and proteins shed by tumors into the blood circulation 13-17. Although blood-based LBx-guided personalized therapy has already entered clinical practice for the management of several cancers 18,19, limited progress in the clinical use of blood-based LBx has been achieved for brain cancer. One major challenge is that the presence of brain tumor biomarkers in the blood circulation is quite limited due to the blood-brain barrier (BBB). The BBB is a unique vascular structure in the brain that prevents molecules from non-selectively crossing between the circulating blood and the extracellular fluid of the central nervous system. The BBB protects neural tissues from being exposed to toxins in the blood circulation, but it also hinders the release of brain tumor-derived molecular biomarkers into the bloodstream, resulting in extremely low concentrations of circulating biomarkers 20,21. Though the vasculature of gliomas is generally leaky, the tumor-associated BBB permeability can be highly heterogeneous 22,23. New vessels may maintain healthy BBB properties and tumor cells may infiltrate the healthy parenchyma where the BBB remains intact 24. There is a need to increase the BBB permeability to ensure sufficient biomarker release for blood-based LBx diagnosis. Circulating tumor DNA (ctDNA), which carries information about the dynamics of cancer-specific genetic and epigenetic alternations, is currently the most well-studied and validated biomarker for LBx. Although a number of publications have demonstrated the ability to detect ctDNA in patients with brain cancer, brain tumor-derived ctDNA is generally detected at low abundance and in a limited number of patients, which makes analysis difficult in routine clinical practice 15,21,25,26. With advanced biomarker detection techniques, ctDNA is detectable in >75% of patients with advanced pancreatic, ovarian, colorectal bladder, melanoma, and head and neck cancer, but is detectable in <10% of glioma patients 21. Current strategies all focus on developing advanced, highly sensitive biomarker detection techniques for analyzing the collected specimens, such as droplet digital PCR (ddPCR) 27, optimized next-generation sequencing (NGS) 28, and advanced spectroscopy 29. While these techniques are critical in improving sensitivity to the presence of these sparse circulating biomarkers, there is a critical need of techniques that overcome the biophysical barrier that is responsible for this sparsity.

We developed the "sonobiopsy" technique to advance the diagnosis of brain cancer. Sonobiopsy uses focused ultrasound (FUS) in combination with microbubbles to disrupt the BBB and enhance the release of tumor-derived biomarkers from the FUS-targeted brain location into the blood circulation. FUS has the potential to target any area in the whole brain with high precision (on the order of millimeter) in animal models and humans. FUS combined with microbubbles is known to transiently and locally disrupt the BBB 30-33 for improved brain drug delivery in preclinical tumor models 34-36 and non-tumor studies 37-43 and clinical trials 38,44,45. We introduced the hypothesis that FUS-induced BBB disruption enables "two-way trafficking" between the brain and bloodstream 46-48. While circulating agents can be allowed to enter the brain using FUS-mediated BBB disruption, brain tumor-derived biomarkers can also be released into the blood circulation to improve the sensitivity of blood-based LBx. Sonobiopsy enables spatiotemporally-controlled biomarker detection, which cannot be achieved by conventional blood-based LBx. Blood LBx can reflect the global molecular status, i.e., coexistence of different genotypic profiles, but cannot provide spatially-precise genetic information 49,50. On the other hand, sonobiopsy can release brain tumor-specific biomarkers from precisely defined tumor locations to identify the molecular profile unique to the target site. Meanwhile, many tumor biomarkers have short half-lives in the blood, on the order of 16 minutes to 2.5 hours for ctDNA, due to clearance 51. The blood samples can be collected immediately after the FUS-induced biomarker release, which should minimize the clearance.

Several proof-of-concept studies have shown the feasibility of sonobiopsy to enhance biomarker release from the brain to the blood. Our previous study has proven the concept that sonobiopsy enriched blood specimens with enhanced green fluorescent protein (eGFP) mRNA using GBM mouse models established by the direct implantation of eGFP-transduced glioblastoma cells into the mouse brain 47. We also showed that sonobiopsy enhanced the release of two brain-specific protein biomarkers (glial fibrillary acidic protein and myelin basic protein) using a healthy pig model 48. By retrospectively analyzing blood samples collected from FUS-mediated drug delivery clinical trials, Meng et al. provided preliminary clinical evidence that FUS-induced BBB disruption increased the concentrations of circulating biomarkers (cell-free DNA, neuron-derived extracellular vesicles, and brain-specific protein) 52. Although promising, there is a lack of compelling evidence that demonstrates the capability of sonobiopsy in improving the diagnostic sensitivity of brain tumor-specific genetic mutations compared with conventional blood-based LBx. Meng et al. detected IDH1-R132H mutation in one patient, who was known to harbor the tumor mutation. However, this did not address the critical question of whether sonobiopsy could enhance the sensitivity in the detection of tumor mutations, which was the goal of our study. This evidence is critically needed to support the clinical application of sonobiopsy.

Over the past decade, large-scale DNA sequencing efforts have identified several key genomic alterations for various brain cancers. Since the 2016 World Health Organization classification, the routine diagnostic workup for GBM requires genetic analysis of tissue samples to guide patients' prognosis stratification and treatment decisions 53. Specifically, GBM frequently harbors the epidermal growth factor receptor mutation variant, EGFRvIII 54,55, and the telomerase reverse transcriptase promoter mutation, TERT C228T 16. The EGFRvIII mutation occurs in 30-40% of GBM patients and represents an aggressive subtype of GBM 56-58. The sensitive characterization of an EGFRvIII-positive tumor may inform personalized drug trials where some agents may outperform other drugs 59. TERT promoter mutations occur in 62% of GBM patients and are associated with poor treatment outcome 16,60. This association may enable variants, such as TERT C228T, to be a prognostic biomarker for clinical outcome. Blood-based liquid biopsies have shown promise in detecting glioma-specific alterations, such as EGFRvIII and TERT C228T, for molecular classification of tumors. However, the low detection sensitivity of current assays limits the positive predictive value 61. In this study, we demonstrated that sonobiopsy significantly improved the sensitivity in the detection of EGFRvIII and TERT C228T mutations in ctDNA compared with conventional blood-based LBx using a mouse model of GBM. As the mouse model cannot represent the technical challenge of FUS delivery through the thick human skull, and biomarkers released by sonobiopsy will be far more diluted in humans than in mice, there is a need to develop a large animal model of GBM to characterize the translational implications in humans. We developed a porcine model of GBM and quantified the sensitivity of sonobiopsy in ctDNA mutation detection. Our study showed, for the first time, that sonobiopsy improved the sensitivity in the detection of two tumor-specific mutations in mouse and porcine models of GBM. This work provides convincing evidence that sonobiopsy can achieve minimally invasive, spatiotemporally-controlled, and sensitive detection of GBM mutations.

## Results

### Sonobiopsy enhanced detection of brain tumor-specific mutations in a mouse GBM model

**Figure 1:**
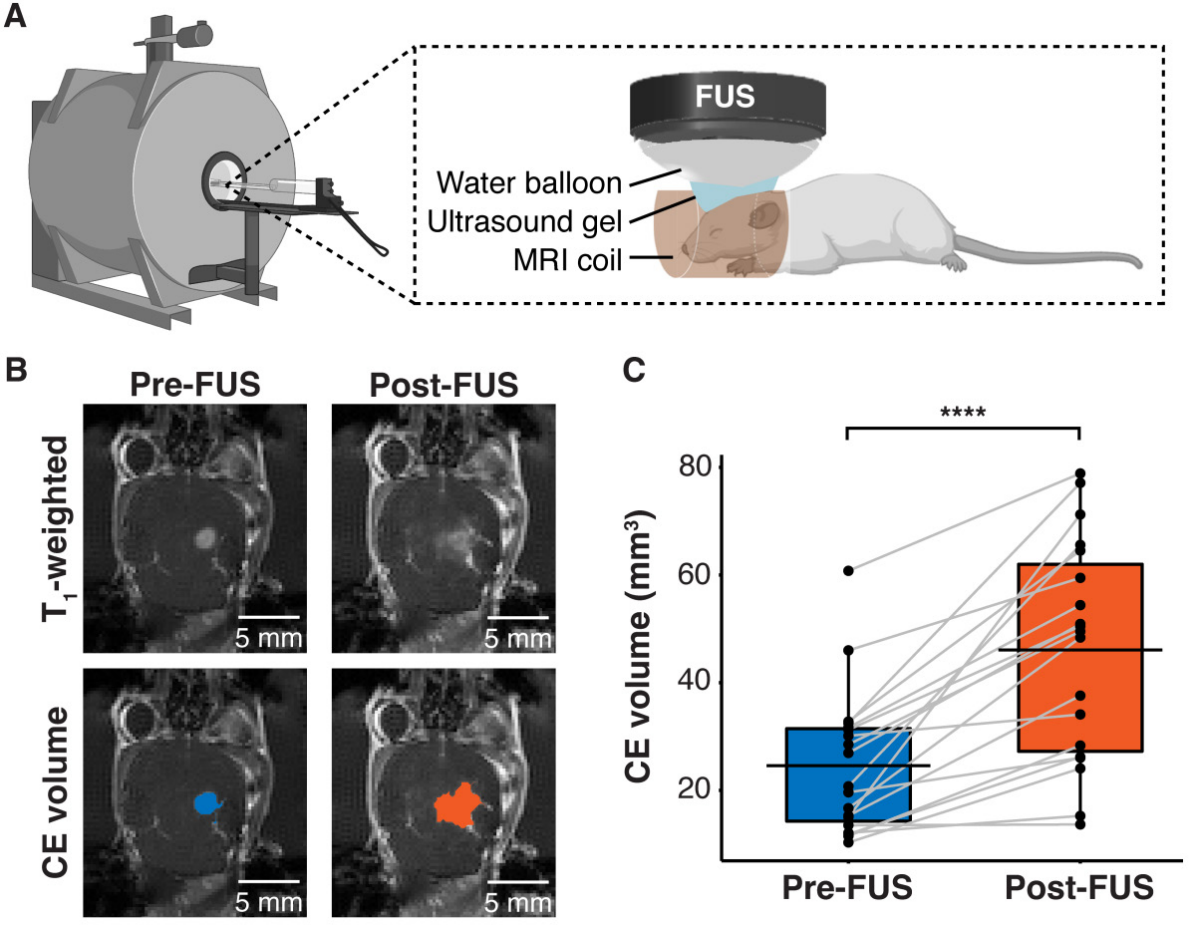
FUS-induced BBB disruption verified by contrast-enhanced T_1_-weighted MRI scans. (*A*) Hardware setup for MRI-guided sonobiopsy in mice. The FUS transducer was coupled with the mouse head using ultrasound gel and a bladder filled with degassed water. (*B*) Contrast-enhanced (CE) T_1_-weighted MRI scans were acquired before FUS to quantify the tumor volume (blue area). Post-FUS MRI scans confirmed FUS-induced BBB disruption (orange area) as an increase in CE volume. (*C*) FUS significantly increased the CE volume (n = 19, *p =* 0.0000038; ^****^p < 0.0001; paired samples Wilcoxon signed rank test) from 24.59 ± 13.21 mm^3^ to 46.09 ± 20.44 mm^3^. Black bars indicate mean in *C*.

Human GBM cells (U87) with EGFRvIII overexpression (U87-EGFRvIII^+^) and carrying TERT C228T mutation were used to establish the mouse model of GBM. This model was used to compare the detection of EGFRvIII and TERT C228T mutations with sonobiopsy or conventional blood-based LBx (blood LBx). A commercially available MRI-compatible FUS system (Image Guided Therapy, Pessac, France) was set up in a small animal MRI scanner (**Figure 1A**). Approximately 10-12 days after intracranial implantation, the mice were assigned to blood LBx (collect blood without FUS) or sonobiopsy (collect blood immediately after FUS). The average tumor volumes for the blood LBx (n = 21) group and the sonobiopsy group (n = 24) were not significantly different (*p* = 0.78; unpaired two-sample Wilcoxon signed rank test) at 25.11 ± 16.25 mm^3^ and 24.59 ± 13.21 mm^3^, respectively. The FUS parameters (FUS pressure and microbubble dose) and post-FUS blood collection time were optimized in a prior parameter optimization study (**Supplementary Figure S1**). Contrast-enhanced (CE) T_1_-weighted MRI scans (**Figure 1B**) were acquired to assess tumor growth and evaluate FUS-induced BBB disruption. FUS significantly increased the volume of tissue with enhanced BBB permeability by approximately 2-fold on average (**Figure 1C**).

**Figure 2:**
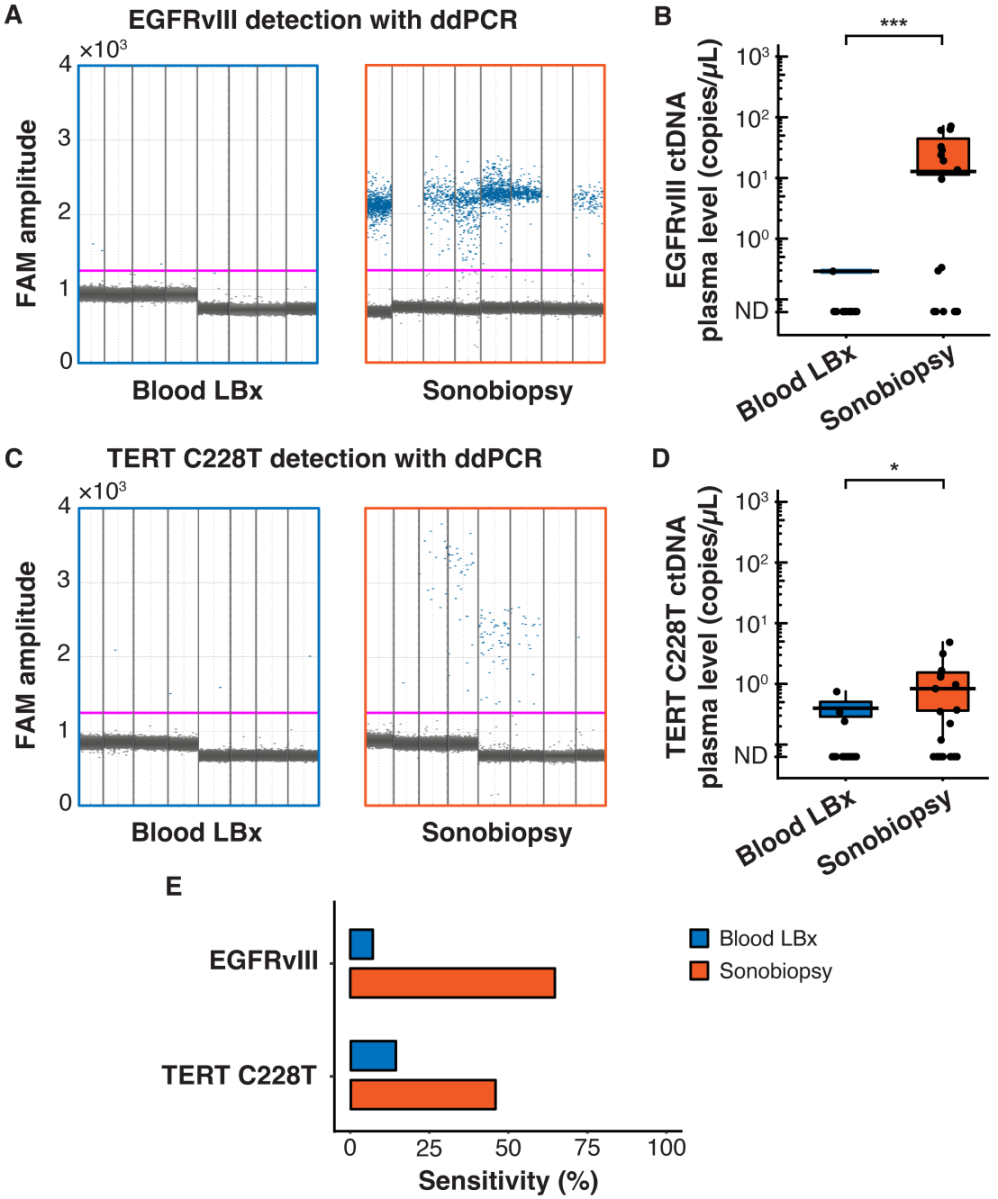
Sonobiopsy increased the sensitivity of EGFRvIII and TERT C228T mutation detections in mouse plasma by ddPCR. (*A*) 1D amplitude plots for blood LBx and sonobiopsy groups demonstrate the detection of EGFRvIII in plasma for each representative subject. The pink line depicts the threshold fluorescence for identifying droplets with positive EGFRvIII expression. (*B*) The level in the sonobiopsy group (n = 17; 19.06 ± 24.74 copies/µL) was significantly greater *(p =* 0.00089; ^***^p < 0.001; unpaired two-sample Wilcoxon signed rank test) compared with the level in the blood LBx group (n = 14; 0.02 ± 0.08 copies/µL). (*C*) 1D amplitude plots for the detection of TERT C228T in plasma for each representative subject in the blood LBx and sonobiopsy groups. The pink line depicts the threshold fluorescence for identifying droplets with positive TERT C228T expression. (*D*) FUS significantly increased the levels of TERT C228T ctDNA in the plasma from 0.06 ± 0.18 copies/µL in the blood LBx group (n = 21) to 0.64 ± 1.19 copies/µL in the sonobiopsy group *(*n = 24;* p =* 0.015; ^*^p < 0.01; unpaired two-sample Wilcoxon signed rank test). (*E*) With ddPCR, sonobiopsy is more sensitive than blood LBx with a detection rate of 64.71% for EGFRvIII and 45.83% for TERT C228T compared with 7.14% and 14.29% for blood LBx, respectively. ND: not detected. Black bars indicate mean in *B* and *D*.

Terminal blood collection via cardiac puncture was performed 10 minutes after FUS sonication. Analysis of the plasma cell-free DNA (cfDNA) found that sonobiopsy enhanced the release of cfDNA compared to conventional blood LBx (**Supplementary Figure S2A**). The plasma levels of mononucleosomal cfDNA (140-230 bp) 51,62-65 increased approximately 2-fold with sonobiopsy (**Supplementary Figure S2B**). Custom ddPCR primers and probes for the detection of EGFRvIII and TERT C228T mutations were validated *in vitro* with cell lines that have known mutation statuses (**Supplementary Figure S3**). The 1D amplitude plots show the detection of EGFRvIII for 8 representative subjects in the blood LBx and sonobiopsy groups (**Figure 2A**). The EGFRvIII ctDNA level in the sonobiopsy group was significantly greater (920-fold) than the blood LBx group (**Figure 2B**). The 1D amplitude plots show the detection of TERT C228T for 8 representative subjects in the blood LBx and sonobiopsy groups (**Figure 2C**). There was a significant increase (10-fold) in the levels of TERT C228T ctDNA with sonobiopsy compared with blood LBx (**Figure 2D**). Sonobiopsy improved the diagnostic sensitivity from 7.14% to 64.71% for EGFRvIII and from 14.29% to 45.83% for TERT C228T (**Figure 2E**). The sensitivity with 95% confidence interval is shown in **Supplementary Table S1**. Taken together, sonobiopsy significantly enhanced the detection of brain tumor-specific mutations.

### No significant off-target tissue damage by sonobiopsy in mouse GBM model

**Figure 3:**
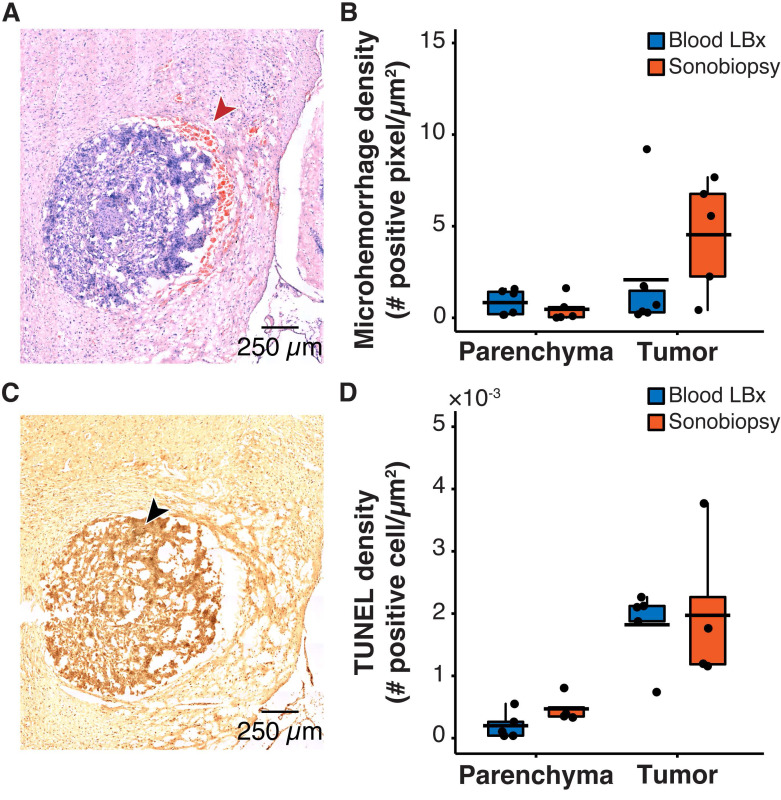
Sonobiopsy did not cause significant acute damage. (*A*) Representative H&E staining for a subject treated with sonobiopsy. The red arrow points to microhemorrhage in the tumor ROI. (*B*) The microhemorrhage density in the parenchyma after sonobiopsy (0.47 ± 0.68 positive pixels/µm^2^, n = 5) was not significantly different compared with that after blood LBx (0.83 ± 0.69 positive pixels/µm^2^; n = 5, *p* = 0.33; unpaired two-sample Wilcoxon signed rank test). There was a nonsignificant increase in microhemorrhage occurrence in the tumor ROI after sonobiopsy (4.54 ± 3.08 positive pixels/µm^2^, n = 5) compared with that after blood LBx (2.08 ± 3.54 positive pixels/µm^2^; n = 5, *p* = 0.18; unpaired two-sample Wilcoxon signed rank test). (*C*) Representative TUNEL staining for a subject treated with sonobiopsy depicts increased apoptotic signal in the tumor ROI. The black arrow points to an apoptotic cell. (*D*) There was no significant difference in TUNEL density for the parenchyma between blood LBx (0.20×10^-3^ ± 0.22×10^-3^ positive cells/µm^2^, n = 5) and sonobiopsy (0.47×10^-3^ ± 0.22×10^-3^ positive cells/µm^2^; n = 5, *p* = 0.11; unpaired two-sample Wilcoxon signed rank test). There was no significant difference in TUNEL density for the tumor ROI between blood LBx (1.82×10^-3^ ± 0.62×10^-3^ positive cells/µm^2^, n = 5) and sonobiopsy (1.97×10^-3^ ± 1.22×10^-3^ positive cells/µm^2^; n = 5, *p* = 0.73; unpaired two-sample Wilcoxon signed rank test). Black bars indicate mean in *B* and *D*.

One safety concern with sonobiopsy was the potential for tissue damage in the parenchyma. H&E staining was performed to quantify the extent of FUS-induced microhemorrhage and TUNEL staining was used to evaluate the number of apoptotic cells. Sonobiopsy led to a non-significant increase in detected microhemorrhage within the tumor region of interest (ROI) (**Figure 3A-B**). There was no off-target damage in the brain parenchyma. Sonobiopsy also did not change the TUNEL expression in the tumor ROI or the brain parenchyma (**Figure 3C-D**).

### Sonobiopsy enhanced detection of brain tumor-specific mutations in a porcine GBM model

**Figure 4:**
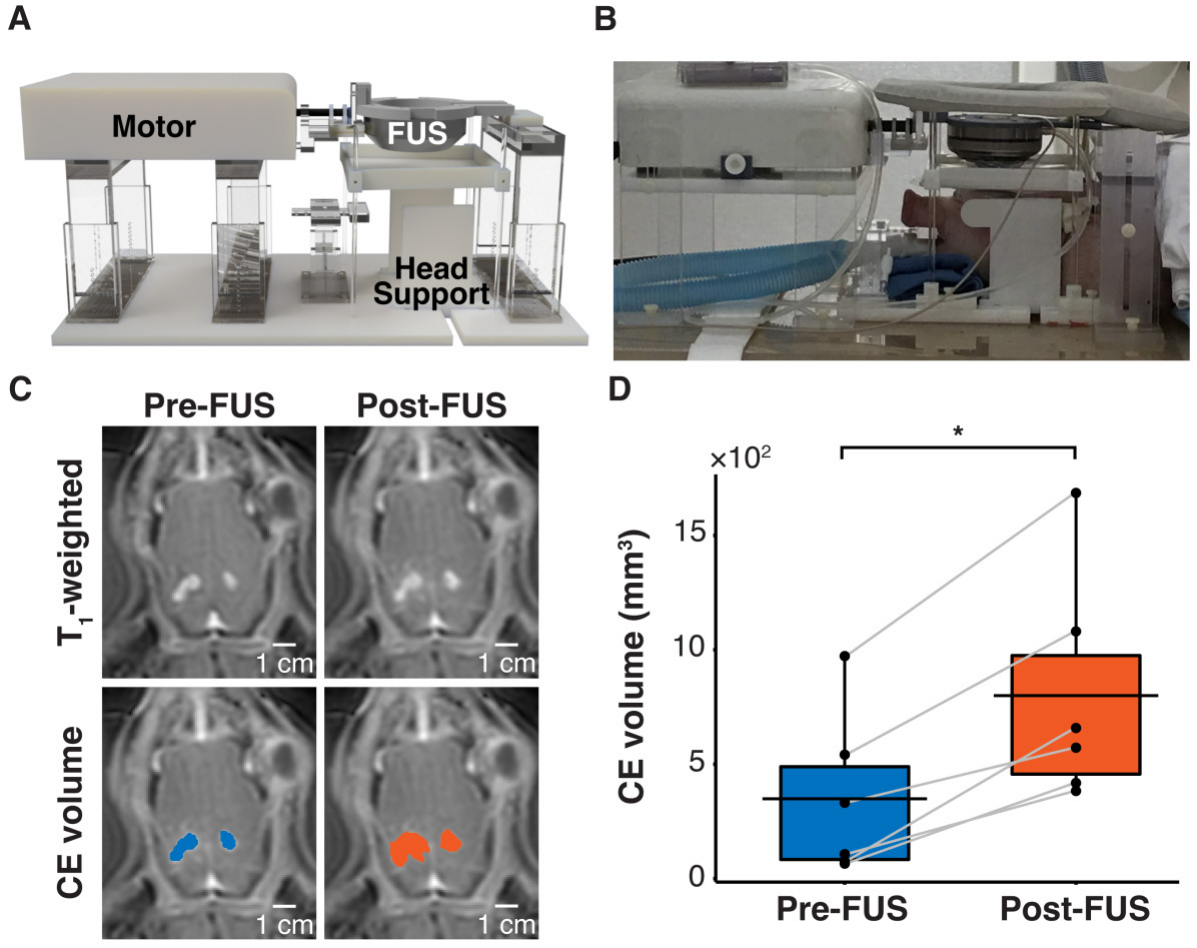
FUS disrupted the BBB in a pig GBM model. (*A*) Hardware setup for MRI-guided sonobiopsy in pigs. The pig head was stabilized by the head supports. The MR-compatible motor enabled the translation of the FUS transducer to specific target locations. (*B*) Placement of pig in sonobiopsy device. (*C*) CE T_1_-weighted MRI scan shows tumor volume (blue area) and FUS-induced BBB disruption (orange area). (*D*) The CE volume significantly increased (n = 6; *p* = 0.031; ^*^p < 0.05; paired samples Wilcoxon signed rank test) from 348.70 ± 358.02 mm^3^ to 799.50 ± 501.19 mm^3^. Black bars indicate mean in *D*.

To validate the clinical translatability of sonobiopsy to enhance the detection of brain tumor-specific mutations, a porcine model of GBM was developed. This model was comprised of a bilateral implantation of the same U87-EGFRvIII^+^ cells as the mouse model in the pig cortex followed by immunosuppressant treatment to prevent rejection of the grafted cells 66,67. The bilateral tumor model capitalized on the unique feature of the large brain volume in pigs and provided the opportunity for sonobiopsy to target two distinct targets in individual pigs. Sonobiopsy was performed approximately 11 days after intracranial implantation. A customized MRI-guided FUS device was developed to sonicate each large animal tumor sequentially (1 hour delay to minimize cross-contamination from biomarker release of the first sonication) in a clinical MRI scanner 48 (**Figure 4A-B**). Contrast-enhanced T_1_-weighted MRI scans confirmed successful BBB disruption of both tumors (**Figure 4C**), where the total CE volume significantly increased post-FUS (**Figure 4D**).

**Figure 5:**
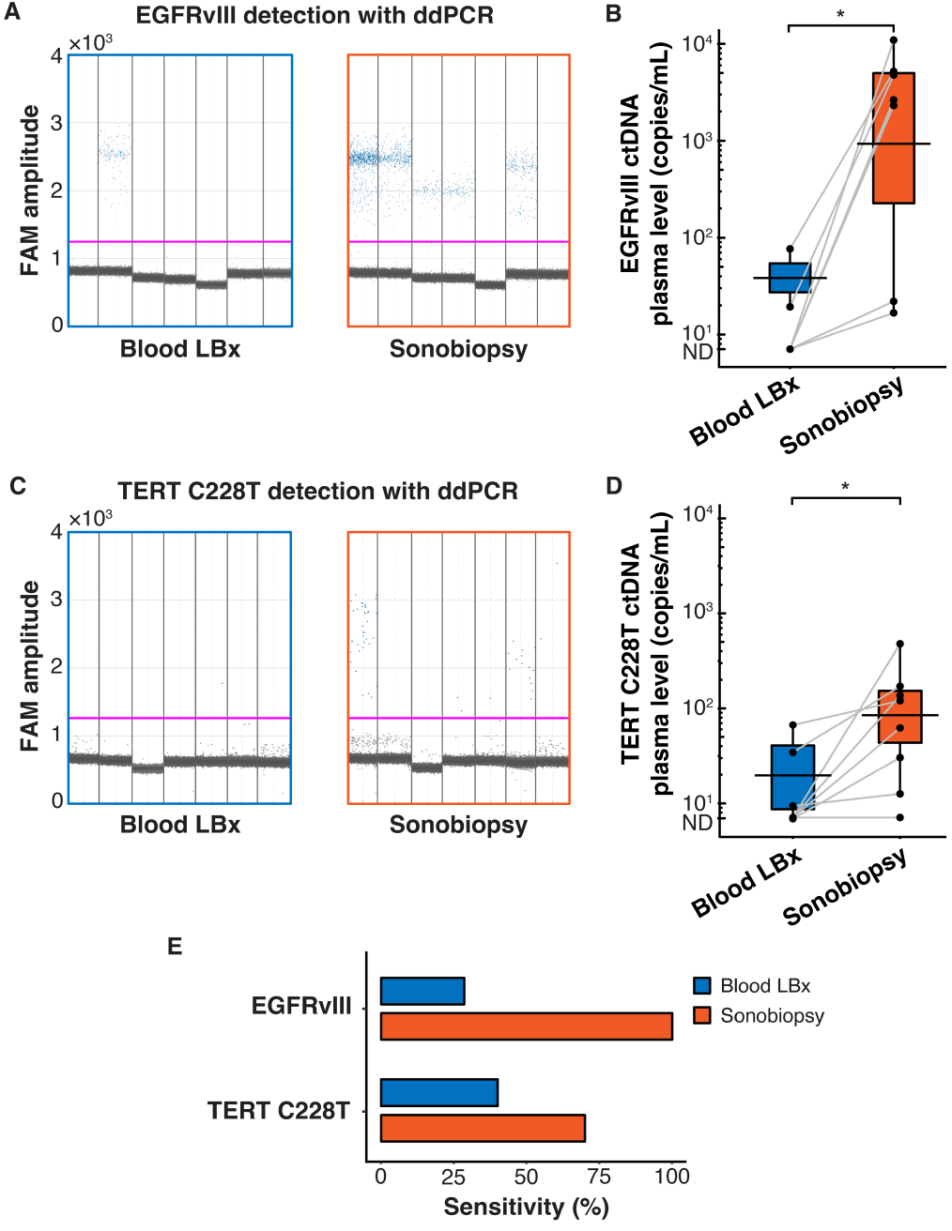
Sonobiopsy increased the sensitivity of EGFRvIII and TERT C228T mutation detections in pig plasma by ddPCR. (*A*) 1D amplitude plots for EGFRvIII detection in plasma for each subject. (*B*) Sonobiopsy significantly increased plasma levels of EGFRvIII ctDNA (n = 7; *p* = 0.016; ^*^p < 0.05; paired samples Wilcoxon signed rank test) from 13.69 ± 28.62 copies/mL to 3697.54 ± 3780.61 copies/mL. (*C*) 1D amplitude plots for TERT C228T detection in plasma for each subject. (*D*) Sonobiopsy significantly increased the plasma levels of TERT C228T ctDNA (n = 10; *p* = 0.022; ^*^p < 0.05; paired samples Wilcoxon signed rank test) from 13.07 ± 23.08 copies/mL to 112.25 ± 150.75 copies/mL. (*E*) With ddPCR, sonobiopsy is more sensitive than blood LBx with a detection rate of 100% for EGFRvIII and 71.43% for TERT C228T compared with 28.57% and 42.86% for blood LBx, respectively. ND: not detected. Black bars indicate mean in *B* and *D*.

Blood samples (5 mL) were collected immediately before and 10 minutes after FUS sonication of each tumor. The ddPCR 1D amplitude plots demonstrate the detection of EGFRvIII for all subjects in the blood LBx (pre-FUS) and sonobiopsy (post-FUS) groups (**Figure 5A**). Sonobiopsy significantly enhanced the release of EGFRvIII ctDNA into the blood by 270-fold (**Figure 5B**). The 1D fluorescence amplitude plots show the detection of TERT C228T with ddPCR for all subjects in the blood LBx and sonobiopsy groups (**Figure 5C**). The levels of TERT C228T ctDNA significantly increased 9-fold with sonobiopsy (**Figure 5D**). The sonobiopsy-induced release improved the diagnostic sensitivity from 28.57% to 100% for EGFRvIII and from 42.86% to 71.43% for TERT C228T (**Figure 5E**). The sensitivity with 95% confidence interval is shown in **Supplementary Table S1**. Sonobiopsy was shown to significantly enhance the detection of brain tumor-specific mutations in a pig GBM model.

### No significant tissue damage by sonobiopsy in pig GBM model

**Figure 6:**
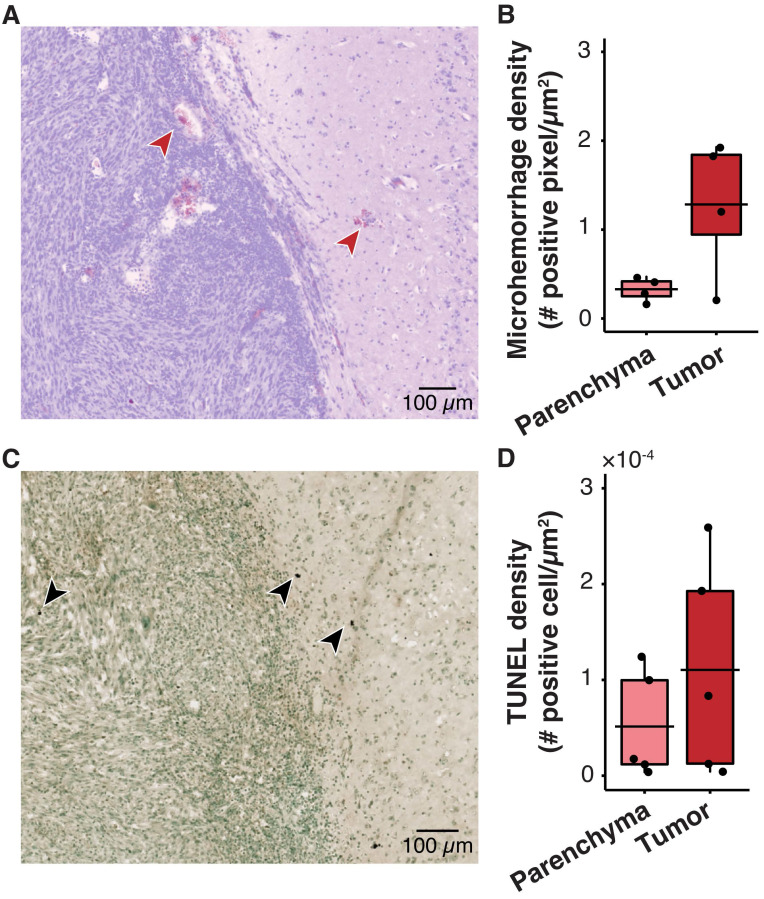
Histological analysis shows no significant tissue damage in pig GBM model. (*A*) Representative horizontal slice with H&E staining. The microhemorrhage occurs in some cases near the edge of the tumor (red arrows). (*B*) Microhemorrhage density was not significantly different between parenchyma (0.33 ± 0.13 positive pixels/µm^2^, n = 4) and tumor (1.28 ± 0.79 positive cells/µm^2^, n = 4, *p* = 0.20; unpaired two-sample Wilcoxon signed rank test). (*C*) Representative TUNEL staining depicts the apoptotic cells (black arrows). (*D*) There was no significant difference between the TUNEL density in the tumor (110.40×10^-4^ ± 112.25×10^-4^ positive cells/µm^2^, n = 4) compared with that in the parenchyma (51.34×10^-4^ ± 56.12×10^-4^ positive cells/µm^2^; (n = 4, *p* = 0.55; unpaired two-sample Wilcoxon signed rank test). Black bars indicate mean in *B* and* D*.

The safety risks associated with large animal sonobiopsy were evaluated by histological staining with H&E and TUNEL. H&E staining shows the presence of microhemorrhage near the edge of the tumor in some cases (**Figure 6A**). However, there was no significant difference in microhemorrhage density between the sonicated tumor ROI and the unsonicated parenchyma (**Figure 6B**). In addition, the TUNEL staining (**Figure 6C**) suggests there was no significant difference between the number of apoptotic cells in the parenchyma compared with the tumor ROI (**Figure 6D**). MRI was used to evaluate acute tissue damage post-FUS. Abnormalities in the post-FUS T_2_^*^-weighted images, i.e., signal intensity changes, were observed (**Supplementary Figure S4B**). The observed tissue damage was consistent with the reversible damage observed in clinical trials of FUS-induced BBB disruption for brain drug delivery 45,68,69.

## Discussion

This study showed that sonobiopsy enriched the plasma ctDNA and improved the detection sensitivity of two GBM mutations without posing significant safety risks. Findings from this study provide convincing evidence from small and large animal models of GBM that supports the clinical translation of sonobiopsy for spatially targeted and temporally controlled detection of ctDNA. Further, this study suggests that sonobiopsy does not pose a clinical risk for significant microhemorrhage or increase in apoptotic cells.

Sonobiopsy addresses the fundamental challenge of obtaining specimens for the sensitive diagnosis and molecular characterization of brain cancer. Sonobiopsy improved the plasma levels of EGFRvIII ctDNA (920- and 270-fold increases for mice and pigs, respectively) and TERT C228T ctDNA (10- and 9-fold increases for mice and pigs, respectively). Sonobiopsy achieved higher detection rates than conventional blood LBx for EGFRvIII (increased the detection sensitivity by 57.57% for mice and 71.43% for pigs) and TERT C228T (increased by 31.54% for mice and 28.57% for pigs). The enhanced plasma levels and detection rate of EGFRvIII are striking compared to those of TERT C228T. This discrepancy may be attributed to the overexpression of the EGFRvIII mutation in the U87-EGFRvIII^+^ cell line. On the other hand, TERT C228T was only expressed on a single chromosome. As a result, the EGFRvIII biomarkers may experience a larger accumulation during tumor growth and greater release after FUS-mediated BBB disruption compared with TERT C228T biomarkers. By using two biomarkers to represent two different gene mutation expression levels, this study demonstrated the range of potential for sonobiopsy.

This study obtained convincing evidence from both small and large animal models that supports the clinical translation of sonobiopsy. The mouse models, while well-characterized and common due to their ease of genetic manipulation, short breeding times, and evolutionary similarities, lack a gyrencephalic structure and other human-like features that are relevant for sonobiopsy, such as skull thickness, brain volume, and blood volume 70,71. Therefore, the results from the mouse experiments in this study demonstrate the feasibility for improving the detection sensitivity of mutations in ctDNA, but this may not be clinically meaningful. A clinically relevant large animal GBM model would corroborate the conclusion. The spontaneous canine glioma model is an option that has similar tumor initiation and progression as humans in a comparable brain size 70,72. However, the tumor incidence is low and the wide variation in size and location limit the reproducibility of the tumor. A GBM model in immunosuppressed cats has been developed, but the unpredictable tumor growth, small brain size, and unique brain vasculature limit its value in preclinical studies 67. The pig model is unique for its human-like brain size, anatomy, and vasculature. In addition, pigs are less expensive and pose less ethical concerns than a non-human primate 67. However, only one group in the world had reported successful development of a pig GBM model 66,67, which may be due to technical challenges in adapting existing stereotactic devices for tumor cell implantation in the pig brain, the difficulty in achieving adequate immunosuppression, as well as the associated high cost for the surgery and animal care. We overcame these challenges and developed the pig GBM model using our custom U87 cell line. Data obtained using the pig GBM model provide convincing evidence that sonobiopsy improved the sensitivity for the detection of EGFRvIII and TERT C228T mutations in ctDNA compared with conventional blood-based LBx. By utilizing two species of different blood volumes, it is possible to extrapolate for the clinical application of sonobiopsy for detecting tumor mutations. In mice, sonobiopsy enhanced the release of EGFRvIII ctDNA by 920-fold and the release of TERT C228T ctDNA by 10-fold when approximately 30% of the total blood volume was collected (0.5 mL collected from 1.7 mL total). In pigs where approximately 5% of the total blood volume (30 mL collected from 620 mL total) was collected, sonobiopsy enhanced the release of EGFRvIII ctDNA by 270-fold and the release of TERT C228T ctDNA by 9-fold. In humans, approximately 1% of the total blood volume can be collected (50 mL collected from 5000 mL total). Assuming linearity between fold change and the fraction of total blood volume collected, it is estimated that sonobiopsy may increase the release of EGFRvIII ctDNA by 212-fold and TERT C228T ctDNA by 8.5-fold.

The integration of sonobiopsy with advanced blood analysis assays has the promise to provide minimally invasive, spatiotemporal-controlled, and sensitive diagnosis of brain cancer. Compared to completely noninvasive technology for detecting circulating markers *in vivo* 73, sonobiopsy is minimally invasive because it requires venipuncture for intravenous delivery of microbubbles and blood collection. ddPCR is a targeted approach to rapidly detect specific known mutations with high sensitivity and high tissue concordance 74-76. Thus, ddPCR was used in our study to detect ctDNA with *a priori* knowledge of the mutations expressed by the implanted GBM tumors. This assisted in the sensitive detection of mutant ctDNA with specific ddPCR probes. In the clinic, this information may not be known, e.g., if sonobiopsy is performed prior to surgical biopsy or if the tumor evolves over time. Thus, future studies will examine the molecular landscape independent of hotspot mutations with approaches such as whole genome sequencing, next-generation sequencing, or bisulfite sequencing. The advancements of these detection techniques have been improving the sensitivity of blood-based LBx. For example, Nassiri et al. demonstrated that the sensitivity of blood-based LBx to detect glioma-derived ctDNA may improve with cfDNA methylation analysis 13. Despite the advancements in the detection techniques, sonobiopsy provides spatially targeted and temporally controlled sample collection that conventional blood-based LBx cannot offer. When a blood sample is drawn during blood-based LBx, the spatial heterogeneity of the tumor cannot be resolved. However, FUS precisely delivers acoustic energy to a discrete target with a high lateral resolution. The BBB disruption that releases biomarkers are confined within that location. Therefore, sonobiopsy has the potential to provide more granularity in characterizing the tumor heterogeneity by targeting different tumor sites and identifying the molecular profile unique to each spatial location 11,17,77. Meanwhile, the level of circulating biomarkers is determined by a balance between biomarker release and clearance processes 78,79. Sonobiopsy can not only enrich the concentration of circulating biomarkers, but also minimize the effect of clearance by collecting the blood samples immediately after biomarker release.

Sonobiopsy did not pose significant safety risks. Although not statistically significant, the average microhemorrhage and TUNEL densities were higher in the tumor after sonobiopsy than the control group. There was a trend for the pigs where the average microhemorrhage density was higher in the tumor than the parenchyma. In addition, hypointensities that indicate microhemorrhages were observed in the post-FUS T_2_^*^-weighted MR images for pigs. This evidence indicated that FUS-mediated BBB disruption led to tissue damage in the FUS-targeted region with minimal off-target effects in the parenchyma outside the FUS-targeted region. FUS-induced tissue damage has been reported in previous studies where the abnormalities recovered within 4 days in mice 80 and within 1-2 months in humans 68,69. In addition, Meng et al., who published the retrospective study on MR-guided focused ultrasound liquid biopsy observed similar damage during the clinical study that was resolved within 24 hours 45. It is not expected that sonobiopsy would contribute to GBM metastasis. Brain tumors, such as GBM, grow locally and rarely metastasize outside the central nervous system (incident rate: 0.4-0.5%) 14,21,81,82. There have not been any documented cases of metastasis or release of tumor cells in preclinical and clinical studies of FUS-induced BBB disruption. This may be the case because existing assays have low sensitivities compared with advanced techniques that have been developed for the purpose of detecting circulating tumor cells 83,84. Regardless, the release of tumor cells is not likely because FUS is less invasive than invasive procedures, e.g., needle biopsy and laser treatment, which could increase the circulating tumor cells 85. Future studies will be performed to validate the long-term safety of sonobiopsy.

We analyzed the correlations between biomarker release, contrast enhancement, and tissue damage. There was no strong correlation observed in the mouse experiment between microhemorrhage density and EGFRvIII ctDNA plasma level (n = 5, Pearson's correlation coefficient *r* = 0.12, *p* = 0.72), microhemorrhage density and change in CE volume (n = 5, *r* = 0.025, *p* = 0.96), or EGFRvIII ctDNA plasma level and change in CE volume (n = 17, *r* = 0.13, *p* = 0.66). Further, there was no strong correlation observed in the pig experiment between microhemorrhage density and change in EGFRvIII ctDNA plasma level (n = 4, *r* = -0.74, *p* = 0.26), microhemorrhage density and change in CE volume (n = 4, *r* = -0.62, *p* = 0.38), or change in EGFRvIII ctDNA plasma level and change in CE volume (n = 6, *r* = -0.43, *p* = 0.29). The lack of a strong correlation suggests that FUS-induced biomarker release is a complex process that may be affected by many variables and/or a larger sample size is needed to detect these correlations.

Besides neuroimaging and surgically acquired tissue for pathology and molecular profiling, sonobiopsy has the potential to become the third pillar for brain tumor management by substantially advancing brain cancer diagnosis, treatment monitoring, and recurrence detection. This enhanced capability could have an important impact throughout the continuum of patient care. In the early diagnostic phase, sonobiopsy could rapidly determine the molecular profile of suspicious lesions observed on neuroimaging scans without the need for surgery. ctDNA has been identified as a promising biomarker for brain cancer diagnosis. The DNA alterations that drive cancer progression, including mutations, copy number changes, and modifications in key driver genes, are detectable in ctDNA 86. By understanding the genetic alterations early, the cancer can be more effectively managed. There is a reported specificity >99% to distinguish cancer patients from healthy individuals 87,88. Technical improvements of analytical approaches may lower the limit of detection to identify mutations with allele frequency as low as 0.1% 89-91. Despite these metrics that make ctDNA a promising early diagnosis biomarker, the main limitation preventing the use of ctDNA for early diagnosis is that early-stage tumors have a low disease burden and do not shed enough ctDNA 75,86,92. This poor sensitivity (43-50% for stage I non-brain cancers using CancerSEEK 88 or CAPP-Seq 93) is the motivation for sonobiopsy. By improving the sensitivity for ctDNA, sonobiopsy may be the missing key to enable early diagnosis with ctDNA, In a mathematical model of plasma biomarker kinetics, Hori and Gambhir showed that a tumor can grow unnoticed for more than 10 years before it is detectable by current clinical blood assays 94. However, if the biomarker shedding rate, i.e., the number of biomarkers entering the blood circulation, was increased 1000-fold (similar to the 920-fold increase of EGFRvIII ctDNA), the detection time reduces to 5 years. This would be crucial for clinicians to diagnose early-stage tumors and initiate treatment to improve progression-free survival and quality of life. Future studies will be performed to investigate the correlation between tumor volume and sonobiopsy sensitivity to demonstrate the capability of sonobiopsy in early-stage cancer diagnosis. In the treatment phase, sonobiopsy could also enable repeated longitudinal sampling to monitor treatment response. Though surgical tissue biopsy is the gold standard to sample a tumor's genetic information 14,75, it can only be performed once or twice because of the surgical risk associated with intracranial surgery. This precludes the routine interrogation necessary to evaluate treatment response. Cerebrospinal fluid (CSF) is an alternative LBx sampling method that has higher sensitivity than blood LBx 95. However, the invasiveness of repeated CSF sampling, which raises safety concerns and the potential risk for developing serious adverse effects in some patients with brain tumors, such as increased intracerebral pressure, may preclude the utility of CSF-based LBx 96. Moreover, CSF-based LBx may not be feasible for tumors with limited DNA shedding to the CSF (e.g., brain tumors that do not contact a CSF compartment or ventricular space) 97. By enriching the blood with brain tumor-derived biomarkers, sonobiopsy could potentially enable the sensitive molecular characterization of brain cancer for longitudinal clinical monitoring. In the post-treatment phase, sonobiopsy could provide complementary information in situations where assessment based on neuroimaging alone remains challenging (e.g., differentiating treatment-induced pseudoprogression from true relapse) 14,98. In addition, sonobiopsy also could support the investigation of tumor-specific molecular mechanisms driving diseases and accelerate the development of effective therapeutic approaches for brain cancer.

## Materials and Methods

All animal procedures were reviewed and approved by the Institutional Animal Care and Use Committee at Washington University in St. Louis in accordance with the Guide for the Care and Use of Laboratory Animals and the Animal Welfare Act.

### Tumor cell preparation

The U87-EGFRvIII^+^ cells were kindly provided by Dr. Frank Furnari from the University of California-San Diego 99. U87 cells also harbor the TERT C228T mutation 100. U87-EGFRvIII^+^-ZsGreen^+^ cells, used for CTC detection, were generated by transduction of U87-EGFRvIII^+^ cells with the lentiviral construct pCRoatan that contains ZsGreen cDNA 101. Both cell lines were cultured as an adherent monolayer in DMEM supplemented with 10% fetal bovine serum, 2 mmol/L l-glutamine, and 100 units/mL penicillin. They were maintained at 37°C in a humidified CO_2_ (5%) atmosphere and the medium was changed as needed. Prior to implantation, cells were dispersed with a 0.05% solution of trypsin/EDTA and adjusted to concentrations needed for tumor implantation. Approximately 3×10^6^ cells for each tumor were implanted in pigs.

### Mouse model of GBM

Immunodeficient mice (strain: NCI Athymic NCr-nu/nu, age: 6-8 weeks, Charles River Laboratory, Wilmington, MA, USA) were used to generate the xenograft GBM model 47. Briefly, mice were anesthetized and the head was fixed on a stereotactic device for injection of the tumor cells. Cells were injected and the tumor growth was monitored using a dedicated 4.7T small animal MRI system (Agilent/Varian DirectDrive^TM^ console, Agilent Technologies, Santa Clara, CA, USA). Starting at 7 days and continuing every 3 days thereafter, MRI scans were acquired to monitor tumor growth and changes in neuroanatomy. Further information is provided in the supplementary information.

### FUS setup and sonobiopsy procedure for mice

The MRI-compatible FUS transducer (Imasonics, Voray sur l'Ognon, France) was made of a 7-element annular array with a center frequency of 1.5 MHz, an aperture of 25 mm, and a radius of curvature of 20 mm. Transducer details were previously described 102 and are provided in the supplementary information. Briefly, the axial and lateral full width at half maximums (FWHM) of the FUS transducer were 5.5 mm and 1.2 mm, respectively. Pressure values were derated to account for the 18% mouse skull attenuation 103. A catheter was placed in the mouse tail vein for intravenous injection.

Coronal and axial T_2_-weighted MRI scans were acquired to image the mouse head and locate the geometrical focus of the transducer (same parameters as aforementioned T_2_-weighted sequence used to monitor tumor growth). The MRI images were imported to a software program (ThermoGuide, Image Guided Therapy, Pessac, France) to locate the focus of the transducer via 3-point triangulation. The transducer was moved to the tumor center for FUS sonication. A pre-FUS axial T_1_-weighted MRI scan was performed to visualize the tumor-induced BBB permeability (same parameters as aforementioned T_1_-weighted sequence used to monitor tumor growth) after intravenous injection of MR contrast agent gadoterate meglumine (Gd-DOTA; Dotarem, Guerbet, Aulnay sous Bois, France) at a dose of 1 mL/kg diluted 1:1 in 0.9% saline.

Definity microbubbles (Lantheus Medical Imaging, North Billerica, MA, USA) at a dose of 100 µL/kg were injected intravenously to the mice, as determined by the prior parameter optimization study (**Supplementary Figure S1**). FUS sonication started 15 seconds prior to microbubble intravenous injection (frequency: 1.5 MHz, pressure: 1.0 MPa, pulse repetition frequency: 5 Hz, duty cycle: 3.35%, pulse length: 6.7 ms, treatment duration: 3 min). FUS sonication was performed at 3 points, evenly spaced apart by 0.5 mm, to enable coverage of the entire tumor volume.

After sonication, Gd-DOTA was re-injected and a post-FUS axial T_1_-weighted MRI scan was performed (same parameters as pre-FUS T_1_-weighted sequence) to quantify the FUS-induced changes in BBB permeability.

### Porcine model of GBM

Pigs (breed: Yorkshire white, age: 4 weeks, sex: male, weight: 15 lbs., Oak Hill Genetics, Ewing, IL, USA) were implanted with the tumor cells on day 0 with an established protocol 66,67. After the pig was sedated by the Veterinary Surgical Services at Washington University, the head was shaved, prepared for sterile surgery, and immobilized in a stereotactic frame on the operating table. The bite bar and ear bars were positioned to secure the head such that the top of the skull was level with the operating table. A 2-3 cm midline cranial skin incision was made and two 5 mm burr holes were drilled 5 mm posterior from bregma and 7 mm to the subject's right and left from midline without breaking the dura (Dremel, Racine, WI, USA). A 50 µL syringe (Hamilton, Reno, NV, USA) used for tumor cell injection was fixed on the stereotactic frame and positioned in the burr hole with the tip at the dura. The syringe was lowered 9 mm to the injection site and the Micro4 controller (World Precision Instruments, Sarasota, FL, USA) infused 40 µL with a rate of 44 nL/sec. There was a 5-minute delay between infusion completion and needle withdrawal to allow the cells to settle in the tissue and prevent backflow. The burr holes were filled with gel foam and the skin incisions were closed with two layers of sutures. A cyclosporine oral solution (Neoral, Novartis Pharmaceuticals, East Hanover, NJ, USA) was administered (25 mg/kg) twice daily via gavage.

Seven days post-surgery, a contrast-enhanced sagittal T_1_-weighted gradient echo MRI scan (TR/TE: 23/3.03 ms; slice thickness: 0.9 mm; in-plane resolution: 0.94×0.94 mm^2^; matrix size: 192×192; flip angle: 27°) was acquired on the 3T Siemens PRISMA Fit clinical scanner (Siemens Medical Solutions, Malvern, PA, USA) to validate tumor growth. An intravenous catheter was placed in the ear for ease of microbubble and gadolinium injections. During the treatment and MR scans, a pulse oximeter (Nonin 7500FO, Plymouth, MN, USA) monitored blood oxygen levels and pulse rate, while heated blankets were used to regulate the temperature.

### FUS setup and sonobiopsy procedure for pigs

A customized MRI-guided FUS device and an established FUS procedure was used for successful BBB disruption 48. The pig head was fixed in a stereotactic head frame with a bite bar and head supports and coupled with the transducer. The FUS system (Image Guided Therapy, Pessac, France) included an MR-compatible 15-element transducer with a center frequency of 650 kHz, an aperture of 65 mm, and a radius of curvature of 65 mm, and an adjustable coupling bladder. The FUS system was attached to an MR-compatible motor for enhanced targeting precision. The FUS transducer calibration is provided in the supplementary information. Briefly, the *in vivo* acoustic pressure was estimated with the top portion of a harvested *ex vivo* pig skull. The axial and lateral FWHM of the transducer was 3.0 mm and 20.0 mm, respectively.

FUS was performed under MR guidance of the 1.5T Philips Ingenia clinical MR scanner (Philips Medical Systems, Inc., Cleveland, OH, USA). Coronal and axial T_2_-weighted spin echo MR images were acquired to examine the neuroanatomy for treatment planning (TR/TE: 1300/130 ms; slice thickness: 1.2 mm; in-plane resolution: 0.58×0.58 mm^2^; matrix size: 448×448; flip angle: 90°). Coronal and axial T_2_^*^-weighted gradient echo MR scans were used to visualize the presence of air bubbles in the acoustic coupling media (TR/TE: 710/23 ms; slice thickness: 2.5 mm; in-plane resolution: 0.98x0.98 mm^2^; matrix size: 224x224; flip angle 18°). The FUS targeting was performed with the same ThermoGuide workflow as the mouse sonobiopsy. Gadobenate dimeglumine (Gd-BOPTA; Multihance, Bracco Diagnostics Inc., Monroe Township, NJ, USA) was intravenously injected at a dose of 0.2 mL/kg and an axial T_1_-weighted ultrafast spoiled gradient echo MR scan was acquired as a pre-FUS baseline (TR/TE: 5/2 ms; slice thickness: 1.5 mm; in-plane resolution: 0.68x0.68 mm^2^; matrix size: 320x320; flip angle 10°).

Definity microbubbles (Lantheus Medical Imaging, North Billerica, MA, USA) at a dose of 20 µL/kg were injected intravenously. FUS sonication started 15 seconds prior to microbubble intravenous injection using the following parameters: frequency: 0.65 MHz, pressure: 3.0 MPa (measured in water; 2.0 MPa measured with the *ex vivo* pig skull), pulse repetition frequency: 1 Hz, duty cycle: 1%, pulse length: 10 ms, treatment duration: 3 min. The bolus injection was determined by the precedence set by the clinical papers that have a similar injection paradigm 45,68,69,104 and the observation that the contrast enhancement via bolus is greater than the enhancement via infusion 105. The 3-minute sonication was previously determined as the time point when all the microbubbles were depleted, as observed by a lack of stable cavitation during passive cavitation detection. The treatment was repeated at 4 individual points spaced 3 mm apart to ensure coverage of the tumor.

After FUS sonication was completed, Gd-BOPTA was intravenously injected and an axial T_1_-weighted MR scan was acquired (same parameters as the pre-FUS T_1_-weighted sequence) to assess the BBB permeability. Coronal T_2_^*^-weighted images were acquired (same parameters as pre-FUS) to assess the potential for FUS-induced tissue damage.

### Mouse and pig plasma isolation

Mouse whole blood (~500 µL) was collected via cardiac puncture and pig whole blood (~10 mL) was collected via percutaneous catheter within peripheral vessel using BD Vacutainer K_2_ EDTA tubes (Becton Dickinson, Franklin Lakes, NJ, USA). Within 4 hours of collection, samples were centrifuged at 3000×*g* for 10 minutes at 4°C to separate the plasma from the hematocrit. Plasma aliquots were put on dry ice immediately for snap freezing and stored at -80°C subsequently for later downstream analysis.

### Cell-free DNA extraction and quantification

Plasma/Serum RNA/DNA Purification Mini Kit (Norgen Biotek, Thorold, ON, Canada) and Plasma/Serum cfc-DNA/cfc-RNA Advanced Fractionation Kit (Norgen Biotek, Thorold, ON, Canada) were used to extract cfDNA from mouse and pig plasma per manufacturer's protocol. cfDNA were eluted in 20 µL of each corresponding buffer and were quantified using Qubit Fluorometric Quantitation (Thermo Fisher Scientific, Carlsbad, CA, USA). The 2100 Bioanalyzer (Agilent Technologies, Palo Alto, CA, USA) was used to assess size distribution and concentration of cfDNA extracted from plasma samples. The total cfDNA concentration was determined with the software as the area under the peaks in the mononucleosomal size range (140-230 bp).

### Cell-free DNA pre-amplification

An initial preamplification reaction was run prior to ddPCR in the case of very low DNA concentration. cfDNA were pre-amplified using Q5 hot start high-fidelity master mix (New England Biolabs, Beverly, MA, USA) with forward and reverse primer pair for EGFRvIII and TERT C228T (same primers used for ctDNA analysis). Pre-amplification was performed with the Eppendorf Mastercycler: 98°C for 3 min; 12 cycles of 98°C for 30 s, 60°C for 1 min; a final extension of 72°C for 5 min, and 1 cycle at 4°C infinite. Preamplified products were directly used for further ddPCR reactions.

### Plasma ctDNA analysis with ddPCR

Custom sequence-specific primers and fluorescent probes were designed and synthesized for EGFRvIII and TERT C228T detection (Sigma Aldrich, St. Louis, MO, USA). The forward and reverse primer sequences for EGFRvIII are 5'-GGCTCTGGAGGAAAAGAAAGGTAATT-3' and 5'-CCTTCGCACTTCTTACACTTGC-3', respectively. The EGFRvIII probe sequence is 5'-CAGATCACGGCTCGTGCGTCCGAGCC-3' with the 6-carboxyfluorescein (6-FAM) fluorophore and the Black Hole Quencher 1 (BHQ1). The forward and reverse primer sequences for EGFRwt are 5'-TCTCAGCAACATGTCGATGGAC-3' and 5'-AGTTCTCCTCTCCTGCACC-3', respectively. The EGFRwt probe sequence is 5'-CTCCCATTGGGACAGCTTGGATCACAC-3' with the hexachlorofluorescein (HEX) fluorophore. The forward and reverse primer sequences for TERT C228T mutant are 5'-CGTCCTGCCCCTTCACCTTC-3' and 5'-GCAGCGCTGCCTGAAACTCG-3', respectively. The TERT C228T mutant probe sequence is 5'-CGTCCCGACCCCTTCCGGGT-3' with 6-FAM and BHQ1. The forward and reverse primer sequences for TERT C228T wild type are the same as those for TERT C228T mutant. The TERT C228T wild type probe sequence is 5'-CGTCCCGACCCCTCCCGGGT-3' with HEX and BHQ1.

ddPCR reactions were conducted using Bio-Rad Q200X according to the manufacturer's instructions (Bio-Rad, Hercules, CA, USA). ddPCR reactions were prepared with 2× ddPCR Supermix for probes (no dUTP) (Bio-Rad, Hercules, CA, USA), 2 µL of target DNA product, of 0.1µM forward and reverse primers, and of 0.1µM probes. For TERT C228T reaction mix, 100µM 7-deaza-dGTP (New England Biolabs, Beverly, MA, USA) was added to improve PCR amplification of GC rich regions in TERT promoter. The QX200 manual droplet generator (Bio-Rad, Hercules, CA, USA) was used to generate droplets. The PCR step was performed on a C1000 Touch Thermal Cycler (Bio-Rad, Hercules, CA, USA) by use of the following program: 1 cycle at 95°C for 10 min, 48 cycles at 95°C for 30 s and 60°C for 1 min, 1 cycle at 98°C for 10 min, and 1 cycle at 12°C for 30min, 1 cycle at 4°C infinite, all at a ramp rate of 2°C/s. All plasma samples were analyzed in technical duplicate or triplicate based on sample availability. Data were acquired on the QX200 droplet reader (Bio-Rad, Hercules, CA, USA) and analyzed using QuantaSoft Analysis Pro (Bio-Rad, Hercules, CA, USA). All results were manually reviewed for false positive and background noise droplets based on negative and positive control samples. Assays were considered positive if >3 droplets exceeded the threshold fluorescence 106,107. Otherwise, the specimen was determined to have 0 copies/µl. EGFRvIII and TERT C228T ctDNA concentrations (copies/µl plasma) were calculated by multiplying the concentration (provided by QuantaSoft) by elution volume, divided by the input plasma volume used during DNA extraction. A subject had a positive detection of the mutation when the levels of mutant ctDNA were >0 copies/μL. The EGFRvIII and TERT C228T sensitivities were calculated as the true positive rate, i.e., number of true positives divided by the sum of true positives and false negatives. The 95% confidence intervals were calculated according to the familiar, asymptotic Gaussian approximation 1.96√p(1-p)/n, where p represents sensitivity and n was the sample size 108,109.

### MRI analysis

MRI processing and analysis was performed using a custom MATLAB script as previously described 48. Further information is provided in the supplementary information.

### Histological analysis

After blood collection, mice were transcardially perfused with 0.01 M phosphate-buffered saline (PBS) followed by 4% paraformaldehyde. Brains were harvested and prepared for cryosectioning. Pig brains were harvested and fixed in 10% formalin. The brains were horizontally sectioned to 15 μm slices and used for H&E staining to examine red blood cell extravasation and cellular injury or TUNEL staining to evaluate number of apoptotic cells. The brain slices were digitally acquired with the Axio Scan.Z1 Slide Scanner (Zeiss, Oberkochen, Germany). QuPath v0.2.0 110 was used to detect areas of microhemorrhage and TUNEL expression. The imaged slice for mouse histological analysis was segmented into the tumor region of interest (ROI) that includes the tumor mass and extends 0.5 mm into its periphery, which is consistent with the safety objectives from previous studies 111 and the potential damage caused by the external and lumen diameters of a biopsy needle 112,113. The parenchyma ROI was defined as the whole imaged slice without the tumor ROI. The tumor ROI for the histological analysis in pigs included the tumor mass and a 3 mm margin 114.

After color deconvolution (hematoxylin vs. eosin), areas of microhemorrhage were detected using the positive-pixel count algorithm. The microhemorrhage density was calculated as the percentage of positive pixel area over the total stained area in the respective ROI. The number of apoptotic cells were detected using the positive cell detection algorithm. The TUNEL density was calculated as the percentage of positive cells over the total stained cells in the respective ROI.

### Statistical analysis

To analyze significance across multiple comparisons, the Kruskal-Wallis test and post hoc Dunn's test with Bonferroni correction was performed (**Figure S1*A*-*F*, *H*,** and ***I***). Where appropriate, the data was analyzed with the paired samples Wilcoxon signed rank test (**Figure 1*C*, 4*D*, 5*B*,** and **5*D***) or the unpaired two-sample Wilcoxon signed rank test (**Figure 2*B*, 2*D*, 3*B*, 3*D*, 6*B*, 6*D*,** and** S2*B***). The correlations between biomarker release, contrast enhancement, and tissue damage were evaluated with the Pearson correlation test. Statistical differences were considered significant (^*^) when *p* < 0.05, (^**^) when *p* < 0.01, (^***^) when *p* < 0.001, and (^****^) when *p* < 0.0001. Descriptive statistics is represented as mean ± SD.

## Supplementary Material

Supplementary figures and table.

## Abbreviations

BBB: blood-brain barrier
CE: contrast-enhanced
cfDNA: cell-free DNA
CSF: cerebrospinal fluid
ctDNA: circulating tumor DNA
ddPCR: droplet digital PCR
eGFP: enhanced green fluorescent protein
EGFRvIII: epidermal growth factor receptor variant III
FUS: focused ultrasound
Gd-BOPTA: gadobenate dimeglumine
Gd-DOTA: gadoterate meglumine
GBM: glioblastoma multiforme
H&E: hematoxylin and eosin
LBx: liquid biopsy
MRI: magnetic resonance imaging
NGS: next-generation sequencing
ROI: region of interest
TERT: telomerase reverse transcriptase
TUNEL: Terminal-deoxynucleotidyltransferase mediated dUTP nick end labelling
