# Supplementary material for: Sonobiopsy for minimally invasive, spatiotemporally-controlled, and sensitive detection of glioblastoma-derived circulating tumor DNA
Source: Theranostics. 2022 Jan 1;12(1):362–78. doi: 10.7150/thno.65597 (PMC8690937; doi:10.7150/thno.65597)
Supplement: Supplementary file 1 — Supplementary figures and table. [file thnov12p0362s1.pdf]

Sonobiopsy for minimally invasive, spatiotemporally-controlled, and sensitive detection of glioblastoma-derived circulating tumor DNA

Christopher P. Pacia[1#], Jinyun Yuan[1#], Yimei Yue[1], Lu Xu[1], Arash Nazeri[2], Rupen Desai[3,4], H. Michael Gach[1,2,5], Xiaowei Wang[6,7], Michael R. Talcott[8], Aadel A. Chaudhuri[5,9,10,11], Gavin P. Dunn[3,4], Eric C. Leuthardt[1,3,12,13], and Hong Chen[1,5*]

1. Department of Biomedical Engineering, Washington University in St. Louis, Saint Louis, MO 63130, USA

2. Mallinckrodt Institute of Radiology, Washington University School of Medicine, Saint Louis, MO, 63110, USA

3. Department of Neurosurgery, Washington University School of Medicine, St. Louis, MO, 63110, USA

4. Andrew M. and Jane M. Bursky Center for Human Immunology and Immunotherapy Programs, Washington University School of Medicine, St. Louis, MO, 63110, USA

5. Department of Radiation Oncology, Washington University School of Medicine, Saint Louis, MO 63108, USA

6. Department of Pharmacology and Regenerative Medicine, University of Illinois at Chicago, Chicago, IL, 60612, USA

7. University of Illinois Cancer Center, Chicago, IL, 60612, USA

8. Division of Comparative Medicine, Washington University School of Medicine, Saint Louis, MO, 63110, USA

9. Department of Genetics, Washington University School of Medicine, St. Louis, MO, 63110, USA

10. Department of Computer Science and Engineering, Washington University in St. Louis, Saint Louis, MO 63130, USA

11. Siteman Cancer Center, Washington University School of Medicine, St. Louis, MO, 63110, USA

12. Department of Neuroscience, Washington University School of Medicine, Saint Louis, MO, 63110, USA

13. Center for Innovation in Neuroscience and Technology, Washington University School of Medicine, Saint Louis, MO, 63110, USA

[#] These two authors contributed equally to this study.

* Address correspondence to Hong Chen, Ph.D. Department of Biomedical Engineering and Radiation Oncology, Washington University in St. Louis. 4511 Forest Park Ave. St. Louis, MO, 63108, USA. Email: hongchen@wustl.edu

Supplementary Methods

FUS metrology. The output of the FUS transducer was calibrated using a piezoelectric hydrophone (Onda HGL-0200, Sunnyvale, CA, USA) in a water tank with an *ex vivo* pig skull. The FUS transducer was fixed in the tank and the hydrophone was attached to a motor. A marker was used to indicate the approximate tumor location (5 mm posterior from bregma, 5 mm from midline) on the top portion of a harvested *ex vivo* pig skull. The skull was secured in the water tank where the marked point was coaxially aligned with the transducer. The axial position of the skull was adjusted such that the geometric focus of the transducer would target the tumor implanted depth (9 mm from bottom face of skull). Acoustic absorbers were lined in the tank to minimize reflections. The focal point of the FUS transducer was identified by raster scanning the hydrophone in the axial and lateral plans. The peak negative pressures under different input levels were calculated. The axial and lateral full width at half maximums (FWHM) were measured along the axial and lateral directions, respectively.

Mouse model of GBM. Immunodeficient mice (strain: NCI Athymic NCr-nu/nu, age: 6-8 weeks, Charles River Laboratory, Wilmington, MA, USA) were used to generate the xenograft GBM model [Ref. 47 in manuscript]. Briefly, mice were anesthetized and the head was fixed on a stereotactic device. A sagittal incision was made with a scalpel and a small hole was created using a micro-drill at 2.0 mm posterior to bregma and 1.5 mm to the subject's right from midline. A total of 2.0×10^4 U87-EGFRvIII$^+$ cells in a volume of 5 μL were injected at a depth of 3.5 mm from the dura using a sterile Hamilton syringe at a rate of 33 nL/sec. The growth of the tumor was monitored using a dedicated 4.7T small animal MRI system (Agilent/Varian DirectDrive^{TM} console, Agilent Technologies, Santa Clara, CA, USA). Starting at 7 days and continuing every 3 days thereafter, MRI scans were acquired to monitor tumor growth and changes in neuroanatomy. The mice were anesthetized under 2% isoflurane and placed in a small animal cradle with an MRI saddle coil (Image Guided Therapy, Pessac, France) and stabilized with a bite bar and ear bars. The rectal temperature was monitored throughout the experiment and maintained at 37°C. During each imaging session, axial T_1-weighted gradient echo (repetition time (TR): 108 ms; echo time (TE): 4 ms; slice thickness: 0.5 mm; in-plane resolution: 0.25×0.25 mm^2; matrix size: 128×128; flip angle: 60°; averages: 16) and axial T_2-weighted fast spin echo (TR/TE: 2000/52 ms; slice thickness: 0.5 mm; in-plane resolution: 0.25×0.25 mm^2; matrix size: 128×128; flip angle: 90°; averages: 4) images were acquired.

FUS setup for mouse experiment. The annular array design of the FUS transducer allowed it to electronically steer the focus in the axial direction. The transducer was connected to an MRI-compatible piezoelectric motor, allowing the position of the transducer to be mechanically adjusted on the horizontal plane. The output of the FUS transducer was calibrated using a hydrophone (Onda HGL-0200, Sunnyvale, CA, USA). The axial and lateral FWHM of the FUS transducer were 5.5 mm and 1.2 mm, respectively. Pressure values were derated to account for the 18% mouse skull attenuation [Ref. 101 in manuscript]. Ultrasound gel was carefully placed atop the mouse head after the hair was removed with a depilatory cream (Nair, Church & Dwight Co., NJ, USA). Deionized and degassed water filled the FUS transducer's water balloon to ensure sufficient acoustic coupling.

MRI analysis. MRI processing and analysis was performed using a custom MATLAB script as previously described [Ref. 48 in manuscript]. First, two region-of-interests (ROI) were defined in the treated (FUS+) and contralateral (FUS–) hemispheres. In the case of tumor pigs, the FUS– area was selected from the parenchyma. Next, a voxel in the FUS+ ROI was considered to contain a compromised BBB if the voxel intensity was greater than 3× standard deviations above the mean intensity within the FUS– ROI. Then, the volume of contrast enhancement (CE volume) was estimated by calculating the sum of FUS+ voxels for each image slice. The analysis was repeated pre-FUS and post-FUS.

Sonobiopsy parameter optimization. Enhanced green fluorescence protein (eGFP)-transfected GL261 murine glioblastoma cells were intracranially implanted to NIH Swiss mice. Mice were randomly assigned to 10 groups once the diameter of the tumor reached 2 mm in any

direction, approximately 10 days, as measured by contrast-enhanced T1-weighted scans. First, the blood collection time was optimized based on which time point maximized the levels of circulating biomarkers. The first three groups had their blood collected either 10 min, 30 min, or 60 min post-FUS. The optimal time was then used for the rest of the experiment. Second, the optimal FUS pressure was evaluated by finding a compromise between increased concentration in biomarkers and FUS-induced tissue damage. The three groups for varying FUS pressure were sonicated at either 0.5 MPa, 1.0 MPa, or 1.5 MPa. The optimal pressure was selected and used for the remainder of the study. Third, the microbubble dose was optimized by comparing biomarkers level in the blood with FUS-induced tissue damage. The three groups for optimizing microbubble dose were either 8×10^8 MB/mL (1×), 40×10^8 MB/mL (5×), or 80×10^8 MB/mL (10×). The control group received no treatment, but the process of MRI acquisition and blood collection remained consistent.

Plasma mRNA analysis. Depending on the treatment group, blood samples were collected from the heart 10 min, 30 min, or 60 min post-FUS. The methods of quantitative polymerase chain reaction (qPCR) analysis of eGFP mRNA have been described in our previous publication [Ref. 47 in manuscript]. Briefly, RNA was extracted from the plasma samples using miRNeasy serum/plasma kit (Catalog no. 217184, Qiagen, Hilden, Germany) followed by Agencourt RNAClean XP beads (Catalog no. A63987, Beckman Coulter Inc., Indianapolis, IN, USA). Extracted RNA was then converted to cDNA using the Applied Biosystems high-capacity cDNA reverse transcription kit (Catalog no. 4368814, Thermo Fisher Scientific, Waltham, MA, USA). 5.8S rRNA was used as the internal control to normalize the PCR data by calculating cycle threshold change (ΔCT) by subtracting CT of the eGFP (CT,eGFP) by the CT of the housekeeping gene, 5.8s rRNA (CT,5.8S). The relative gene expression level was determined using the 2-ΔCT method.

Supplementary Figures

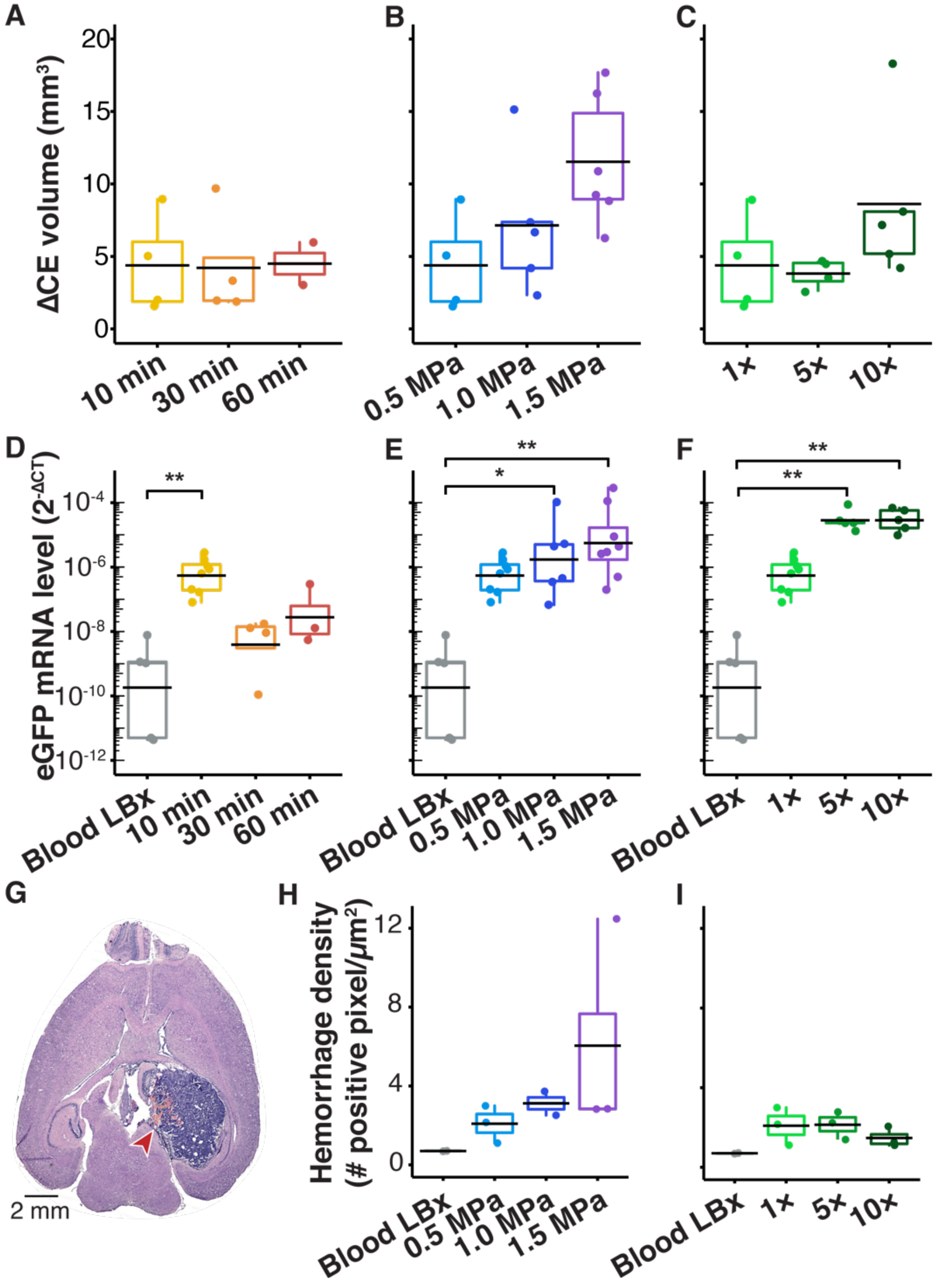

Figure S1. Sonobiopsy parameter optimization by qPCR. Quantification of FUS-induced change in contrast-enhanced volume (ΔCE volume) volume for different blood collection times post-FUS (*A*), different FUS pressures (*B*), and different microbubble doses (*C*). The ΔCE volume was not significantly different between each of the treatment groups compared with one another (Kruskal-Wallis test and post hoc Dunn's test with Bonferroni correction). FUS-induced biomarker release for treatment groups with varying blood collection times (*D*), FUS pressure (*E*), and microbubble dose (*F*). There were significant increases in eGFP mRNA levels at 10-min post-FUS blood collection (474-fold increase; n = 8, $p = 0.0015$; $^{**}p < 0.01$), with FUS pressures of 1.0 MPa (9,694-fold increase; n = 6, $p = 0.03$; $^{*}p < 0.05$) and 1.5 MPa (26,514-fold increase; n = 8, $p = 0.0013$; $^{**}p < 0.01$) and with microbubble doses of 5× (17,790-fold increase; n = 5, $p = 0.0019$; $^{**}p < 0.01$), and 10× (18,208-fold increase; n = 5, $p = 0.0017$; $^{**}p < 0.01$). Statistical analysis was performed with Kruskal-Wallis test and post hoc Dunn's test with Bonferroni correction in comparison with blood

LBx. (*G*) Representative whole-brain horizontal slice shows tumor and hemorrhage. The extent of tissue damage, as indicated by hemorrhage density for each treatment group of varying FUS pressure (*H*) and microbubble dose (*I*). There was no statistical difference between each of the treatment groups (n = 3) from one another (Kruskal-Wallis test and post hoc Dunn's test with Bonferroni correction). Black bars indicate mean in *A*-*F*, *H*, and *I*.

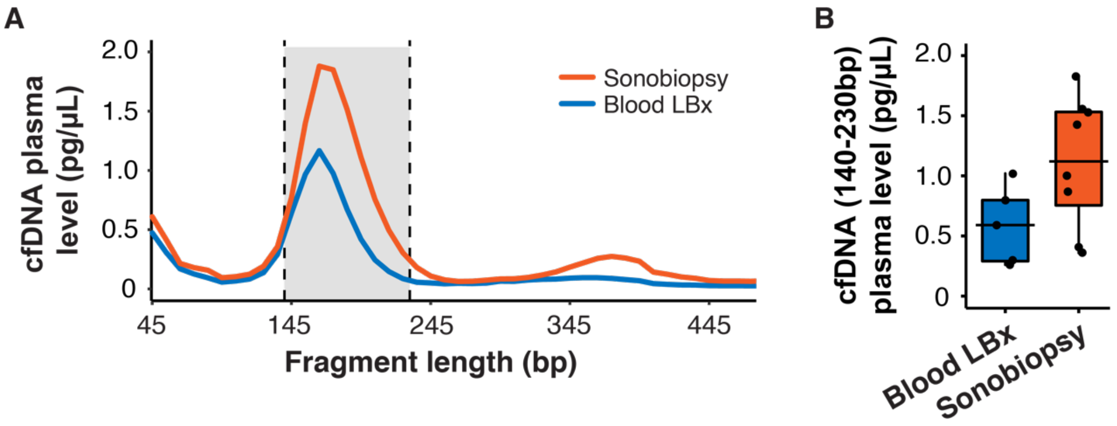

Figure S2. Fragmentation profile of sonobiopsy-released cfDNA. (*A*) The average size distribution of cfDNA fragment levels were compared in the blood collected after FUS for the sonobiopsy group (orange line, n = 8) or without FUS for the blood LBx group (blue line, n = 5). The plasma levels of mononucleosomal cfDNA fragments between 140 and 230 bp (gray dashed area) were quantified. (*B*) Sonobiopsy increased the cfDNA levels from 0.030 ± 0.016 pg/μL to 0.056 ± 0.027 pg/μL (mean ± SD) in the mononucleosomal size range (p = 0.09; unpaired two-sample Wilcoxon signed rank test).

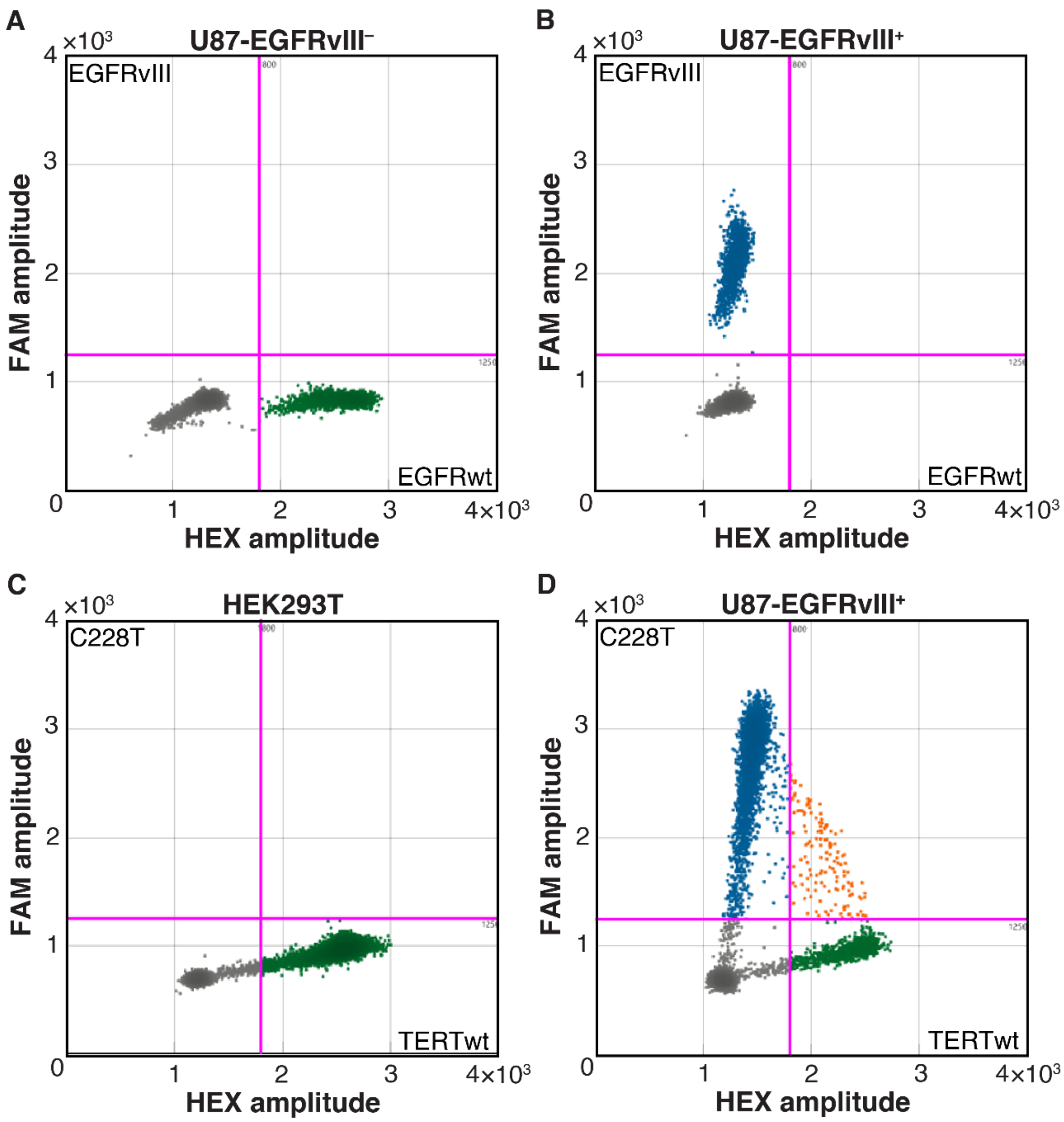

Figure S3. Validation of custom ddPCR primers and probes for detecting EGFRvIII and TERT C228T mutations in vitro. (*A*) Wild type U87 cell line without EGFRvIII expression (U87-EGFRvIII^{-}) had no detectable copies of EGFRvIII. (*B*) U87 cell line transfected with EGFRvIII overexpression (U87-EGFRvIII^{+}) had positive detection of EGFRvIII. (*C*) Human embryonic kidney cells (HEK293T) had no detectable copies of TERT C228T. (*D*) ddPCR detected both TERT C228T and TERT wild type (TERTwt) in U87 cell line with known TERT C228T expression (U87-EGFRvIII^{+}).

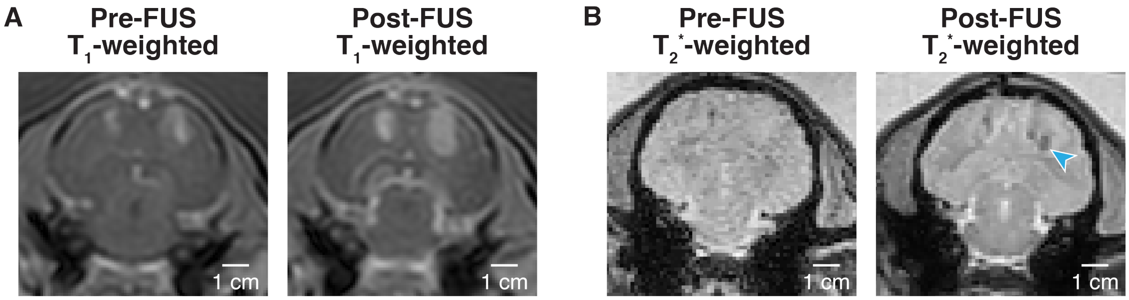

Figure S4. Evaluation of sonobiopsy safety with MRI. (*A*) T_1-weighted MR images confirmed consistent BBB disruption at each tumor in the bilateral pig GBM model. (*B*) Abnormality in T_2^*-weighted MR images (blue arrow) indicative of microhemorrhage was observed at the FUS sonication target site.

Table S1. Sensitivity (±95% confidence intervals) of mutation detection

		Blood LBx	Sonobiopsy
Species	Mutation	Sensitivity %<br>(95% confidence interval)	Sensitivity %<br>(95% confidence interval)
Mouse	EGFRvIII	7.14 ± 13.49	64.71 ± 22.72
Mouse	TERT C228T	14.91 ± 14.97	45.83 ± 19.93
Pig	EGFRvIII	28.57 ± 33.47	100 ± 0
Pig	TERT C228T	42.86 ± 36.66	71.43 ± 33.47
